# Integration of 3D anatomical data obtained by CT imaging and 3D optical scanning for computer aided implant surgery

**DOI:** 10.1186/1471-2342-11-5

**Published:** 2011-02-21

**Authors:** Gianni Frisardi, Giacomo Chessa, Sandro Barone, Alessandro Paoli, Armando Razionale, Flavio Frisardi

**Affiliations:** 1 "Epochè" Orofacial Pain Center, Nettuno (Rome), Italy; 2 Department of Prosthetic Rehabilitation, University of Sassari, Italy; 3 Department of Mechanical, Nuclear and Production Engineering, University of Pisa, Italy

## Abstract

**Background:**

A precise placement of dental implants is a crucial step to optimize both prosthetic aspects and functional constraints. In this context, the use of virtual guiding systems has been recognized as a fundamental tool to control the ideal implant position. In particular, complex periodontal surgeries can be performed using preoperative planning based on CT data. The critical point of the procedure relies on the lack of accuracy in transferring CT planning information to surgical field through custom-made stereo-lithographic surgical guides.

**Methods:**

In this work, a novel methodology is proposed for monitoring loss of accuracy in transferring CT dental information into periodontal surgical field. The methodology is based on integrating 3D data of anatomical (impression and cast) and preoperative (radiographic template) models, obtained by both CT and optical scanning processes.

**Results:**

A clinical case, relative to a fully edentulous jaw patient, has been used as test case to assess the accuracy of the various steps concurring in manufacturing surgical guides. In particular, a surgical guide has been designed to place implants in the bone structure of the patient. The analysis of the results has allowed the clinician to monitor all the errors, which have been occurring step by step manufacturing the physical templates.

**Conclusions:**

The use of an optical scanner, which has a higher resolution and accuracy than CT scanning, has demonstrated to be a valid support to control the precision of the various physical models adopted and to point out possible error sources. A case study regarding a fully edentulous patient has confirmed the feasibility of the proposed methodology.

## Background

Over the last few years, dental prostheses supported by osseointegrated implants have progressively replaced the use of removable dentures in the treatment of edentulous patients. The restoration of missing teeth must provide a patient with aesthetical, biomechanical and functional requirements of natural dentition, particularly concerning chewing functions. When conventional implantation techniques are used, the clinical outcome is often unpredictable, since it greatly relies on skills and experience of dental surgeons.

The placement of endosseous implants is based on invasive procedures which require a long time to be completed. Recently, many different implant planning procedures have been developed to support oral implant positioning. Number, size, position of implants must be related to bone morphology, as well as to the accompanying vital structures (e.g. neurovascular bundles). Complex surgical interventions can be performed using preoperative planning based on 3D imaging. The developments in computer-assisted surgery have brought to the definition of effective operating procedures in dental implantology. Several systems have been designed to guide treatment-planning processes: from simulation environments to surgical fields [1]. The guided approaches are generally based on three-dimensional reconstructions of patient anatomies processing data obtained by either Computed Tomography (CT) or Cone-Beam Computed Tomography (CBCT) [2]. These methodologies allow more accurate assessments of surgical difficulties through less invasive procedures and operating time reductions. In particular, radiographic data (depth and proximity to anatomical landmarks) and restorative requirements are crucial for a complete transfer of implant planning (positioning, trajectory and distribution) to surgical field [3]. Virtual planning processes provide digital models of drill guides, which are typically manufactured by stereo-lithography and used as surgical guidance in the preparation of implant receptor sites.

In the past decade, a methodology based on the use of two different guides and a double CT scan procedure, has been introduced [4] and later commercialized as NobelGuide^® ^by NobelBiocare (Zurich, Switzerland). This procedure involves an intermediate template (*radiographic template*) that is used to refer the soft tissues with respect to the bone structure derived from patient CT scan data. The guide is manufactured on the basis of diagnostic wax-up reproducing the desired prosthetic end result. The diagnostic wax-up is obtained starting from the dental cast, produced from the impression of the patient's mouth, and helps in the definition of a proper dental prosthesis design. Moreover, the radiographic template is made of a non radio-opaque material, usually acrylic resin, to avoid image disturbs when CT scans of patients are carried. Then, the template is separately scanned changing radiological parameters in order to visualize the acrylic resin. The computer-based alignment of the prosthetic model with respect to the maxillofacial structure is obtained by small radio-opaque gutta-percha spheres inserted within the radiographic template. These gutta-percha markers are visible in both the different CT scans and can be used as references to register the two data sets through point-based rigid registration techniques [5].

Specific 3D image-based software programs for implant surgery planning, based on CT scan data, have been recently developed and clinically approved by many manufacturers. These software applications allow surgeons to locate implant receptor sites and simulate implant placement [6]. The planned implant positions are then transferred to the surgical field by means of a surgical guide made by stereo-lithographic techniques. Surgical guides can be bone-supported, tooth-supported or mucosa-supported depending on the specific patient's conditions. Bone-supported guides are designed to fit on the jawbone and can be used for partially or fully edentulous cases, while tooth-supported guides are tailored to fit directly on the teeth. The latters are mostly effective for single tooth and partially edentulous cases. Mucosa-supported surgical guides are rather designed for placement on soft tissues and are recommended for fully edentulous patients when minimally invasive surgery is required.

The surgical guide is then placed within the patient's mouth and can be anchored, especially when mucosa-supported guides are used, to the jawbone by stabilizing pins (Anchor Pins).

The weak point of the whole procedure relies on the accuracy in transferring information deriving from CT data into surgical planning. Geometrical deviations of implant positions between planning and intervention stages could cause irreversible damages of anatomical structure, such as sensory nerves. The surgical guide should closely fit with the hard and/or soft tissue surface in a unique and stable position in order to accurately transfer the pre-operative treatment plan. If the surgical template is not accurate, the fit will be improper, compromising the implant placement. Even small angular errors in the placement of perforation guides can, indeed, propagate in considerable horizontal deviations due to the depth of the implant.

A previous in ex vivo study to assess the accuracy of 10-15 mm-long implant positioning using CBCT, revealed a mean angular deviation of 2° (*SD *± 0.8, range 0.7° ÷ 4°) and a mean linear deviation of 1.1 mm (*SD *± 0.7 mm, range 0.3 ÷ 2.3 mm) at the hexagon and 2 mm (*SD *± 0.7 mm, range 0.7 ÷ 2.4 mm) at the tip [7].

Sarment *et al. *[8] compared the accuracy of a stereo-lithographic surgical template to conventional surgical template *in vitro*. An average linear deviation of 1.5 mm at the entrance, and 2.1 mm at the apex for the conventional template, as compared with 0.9 and 1.0 mm for the stereo-lithographic surgical template was reported.

Di Giacomo *et al. *[9] published a preliminary study involving the placement of 21 implants using a stereo-lithographic surgical template, showing an angular deviation of 7.25° between planned and actual implant axes, whereas the linear deviation was 1.45 mm.

In a recent study [10], the accuracy of a surgical template in transferring planned implant position to the real patient surgery has been assessed. The mean mesio-distal angular deviation of the planned to the actual was 0.17° (*SD *± 5.02°) ranging from 0.262° to 12.2°, though, the mean bucco-lingual angular deviation was 0.46° (*SD *± 4.48°) ranging from 0.085° to 7.67°.

These studies confirm that the error could be high, especially in neurovascular anatomical districts, such as the mandibular nerve. In this anatomical area, a moderate damage may also result in severe symptoms. For example, the lesion of the mandibular nerve is of the Wallerian degenerative type [11], which is a slow degenerative process and the diagnosis by laser-evoked potentials and trigeminal reflexes would allow early decompression [12].

Deviations between planning and postoperative outcome may reflect the sum of many error sources. For instance, CT scan quality and processing of DICOM (*Digital Imaging and Communication in Medicine*) images affect the creation of the corresponding 3D digital models. Misalignment errors can also be introduced during the arrangement of the radiographic template within the maxillofacial structures by the gutta-percha markers. Moreover, further inaccuracies can be introduced in manufacturing physical models by stereo-lithographic techniques.

This paper concerns the development of an innovative methodology to evaluate the accuracy in transferring CT based implant planning into surgical fields for oral rehabilitation.

## Methods

The proposed methodology is based on the combined use of CT scan data and a structured light vision system. In particular, the data acquisition phase regards two different scanning technologies: radiological scanning and optical scanning.

A clinical case, relative to a fully edentulous patient, has been used as test case to assess the feasibility of the proposed methodology. The ethics approval was obtained by Human Research Ethics Committee at the Sassari Hospital (n° 971) and written form approval was obtained by the patient.

### Optical scanning

**Figure 1:**
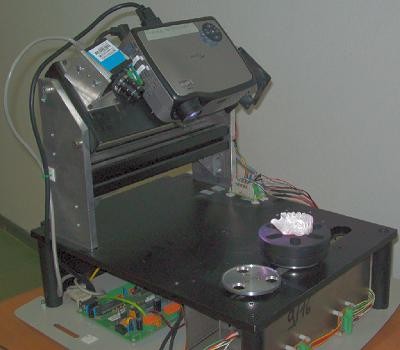
**Optical scanner**. 3D optical scanner used to capture dental models

The 3D optical scanner used in this work is based on a stereo vision approach with structured coded light projection [13]. The optical unit is composed of a monochrome digital camera (CCD - 1280 × 960 pixels) and a multimedia white light projector (DLP - 1024 × 768 pixels) that are used as active devices for a triangulation process. The digitizer is integrated with a rotary axis, automatically controlled by a stepper motor with a resolution of 400 steps per round (Figure 1). The scanner is capable of measuring about 1 million 3D points within the field of view (100 mm × 80 mm), with a spatial resolution of 0.1 mm and an overall accuracy of 0.01 mm [13].

### CT scan data

CT scanning of maxillofacial region is based on the acquisition of several slices of the jaw bone at each turn of a helical movement of an x-ray source and a reciprocating area detector. The acquired data can be stored in DICOM format.

**Figure 2:**
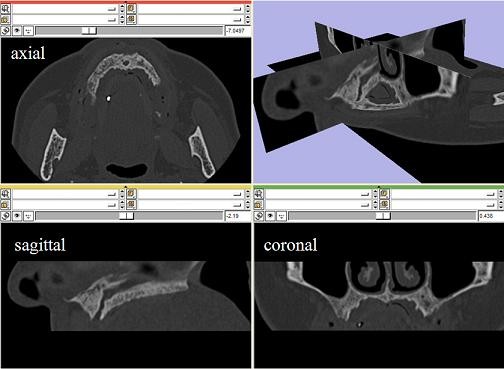
**CT data**. Maxilla CT data in the axial, sagittal and coronal planes and a fully 3D vision

In this work, CT scanning has been performed using a system Toshiba Aquilion by Toshiba Medical Systems, Japan, with 0.5 mm slice thickness. 3D models have been reconstructed processing DICOM images by means of 3D Slicer (version 3.2), a freely available open source software initially developed as a joint effort between the Surgical Planning Lab at Brigham and Women's Hospital and the MIT Artificial Intelligence Lab. The software has now evolved into a national platform supported by a variety of federal funding sources [14]. 3D Slicer is an end-user application to process medical images and to generate 3D volumetric data set, which can be used to provide primary reconstruction images in three orthogonal planes (axial, sagittal and coronal). 3D models of anatomical structure can be generated through a powerful and robust segmentation tool on the basis of a semi-automated approach. The displayed gray level of the voxels representing hard tissues can be dynamically altered to provide the most realistic appearance of the bone structure, minimizing soft tissues and the superimposition of metal artifacts (Figure 2). Initial segmentation of CT data can then be obtained by threshold segmentation. This involves the manual selection of a threshold value that can be dynamically adjusted to provide the optimal filling of the interested structure in all the slices acquired.

### 3D reconstructions

**Figure 3:**
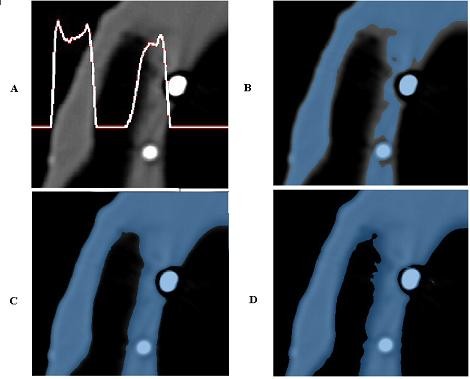
**(A-D) CT data segmentation process**. (A) DICOM image of the radiographic template with associated a row grey intensity level, (B-D) segmentation with three different threshold values.

The accuracy of 3D reconstruction based on CT data analysis may be affected by several factors that should be considered in surgical treatment planning. A reduction of image quality may be caused by metallic artifacts and/or patient motions. Moreover, the influence of an appropriate segmentation on the final 3D representation is a matter of utmost importance [15]. The segmentation process typically relies on the adopted mathematical algorithm, on spatial and contrast resolution of the slice images, on technical skills of the operator in selecting the optimal threshold value. Metal restorations as well as tissues not belonging to the structure of interest (i.e. antagonistic teeth) must be carefully cleaned up from the CT scan images when models for interactive planning are prepared. This process can lead to different volume reconstructions due to the operator's selection of threshold values, even if proved and patented software is used. In particular, the detection of the optimal threshold value is not straightforward when images presenting smooth intensity distributions are processed (Figure 3). For this reason, a methodology to verify the accuracy of the 3D reconstruction of CT derived images would be necessary for clinical applications.

**Figure 4:**
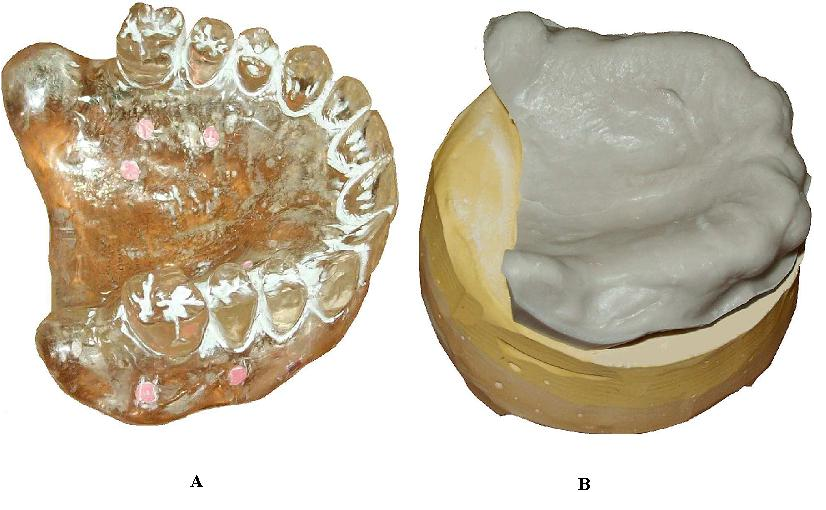
**(A-B) Preoperative and anatomical dental models**. (A) Radiographic template with gutta-percha markers, (B) gypsum dental cast.

**Figure 5:**
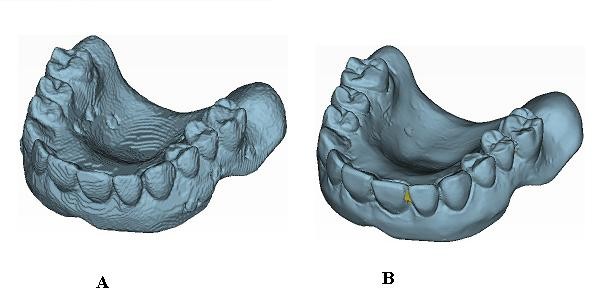
**(A-B) Digital models of the radiographic template**. 3D digital models of the radiographic template obtained by CT data (A) and by the optical scanner (B).

In this work, a validation process for 3D reconstructions of radiographic templates used in implant guided surgery has been developed using the optical scanner. As previously illustrated, the radiological template (Figure 4A) is manually manufactured on the basis of the diagnostic wax-up to take into account prosthesis design, and on the gypsum dental cast (Figure 4B) to assure the optimal fitting of the mating surfaces. The 3D model of the radiographic template is reconstructed processing the DICOM images (Figure 5A). The radiographic template is also acquired by the optical scanner. The 3D model as obtained by the structured light scanning system (Figure 5B) is used as the gold standard to improve the accuracy of the CT reconstruction. The comparison between the CT reconstructed and the optically captured models gives the information to optimize the parameters of the DICOM images segmentation process. The data acquired by the optical scanner are aligned to the model obtained by the CT reconstruction through a point-based registration technique. Correspondent pairs of points are manually selected on the two different models and the rigid transformation between the two objects is determined by applying the singular value decomposition (SVD) method [5]. The alignment is then refined by applying a surface-based registration technique through best fitting algorithms [16].

**Figure 6:**
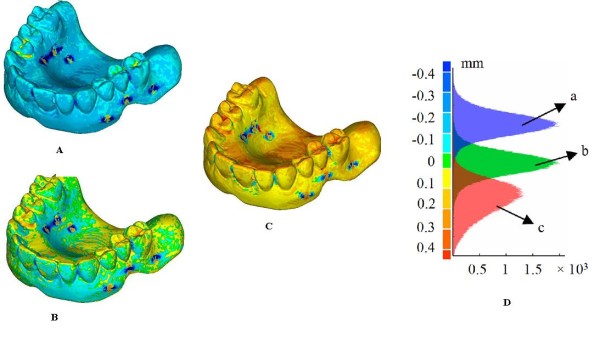
**(A-D) Full-field 3D comparisons of three different reconstructions of the radiographic template**. Full-field 3D compare of three different DICOM reconstructions of the radiographic template with respect to the model obtained by the optical scanner and relative histogram plots (D). The DICOM model (Figure 5A) results smaller (A), comparable (B) and greater (C) than the one obtained by the optical scanner (Figure 5B).

Figure 6 shows the full-field 3D compare of three different reconstructions of the radiological guide, obtained varying the threshold values, with respect to the model obtained by the optical scanner. The distribution of discrepancies between the datasets obtained using the two scanning technologies, with both positive and negative deviations, quantifies the dimensional difference of the CT based reconstruction that can turn out to be smaller (Figure 6A) or greater (Figure 6C). The search of the optimal threshold value can therefore be made by minimizing the absolute mean of the distances between the two models (Figure 6B). Histogram plots of these distributions are reported in Figure 6D, whereas Table 1 reports the associated statistical data (mean and standard deviation).

**Table 1:** Statistical data relative to different DICOM reconstructions

3D Compare	*Mean value* [mm]	*SD* [mm]
A	-0.224	0.226
B	-0.008	0.200
C	0.185	0.179

Mean and standard deviation of the discrepancies in the three different cases reported in Figure 6 and relative to the threshold values used in Figure 3 (B-D)

## Results

**Figure 7:**
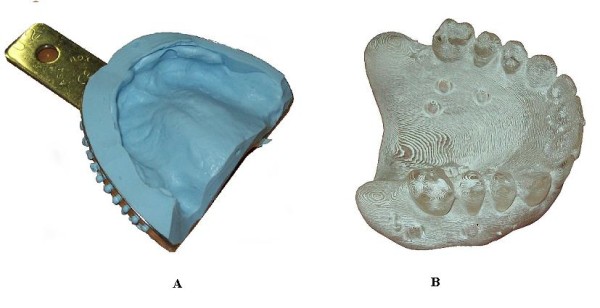
**(A-B) Impression and radiographic template**. Patient's mouth impression (A) and Duplicate Radiographic Template (B)

In the present work, a clinical case, relative to a fully edentulous patient, has been used as test case to assess the accuracy of the various steps concurring in manufacturing surgical guides. A study surgical template (Figure 7B), called Duplicate Radiographic Template (D.R.T) and based on the same CT data used to fabricate the mucosa-supported surgical guide, has been manufactured by a stereo-lithographic process. This template does not present the holes to hold the drill guides since the first requirement was just the reproduction of the only functional areas to wearing the guide. All the physical models (impression, cast, radiographic template, study surgical template) have been acquired by the optical scanner. The 3D digital models have been realigned by best fitting techniques in order to evaluate the discrepancies between the different shapes. The virtual alignments have been conducted by only referring the mating surfaces of the various models, since the crucial problem regards the proper fit between the final surgical guide and the patient's mucosa.

**Figure 8:**
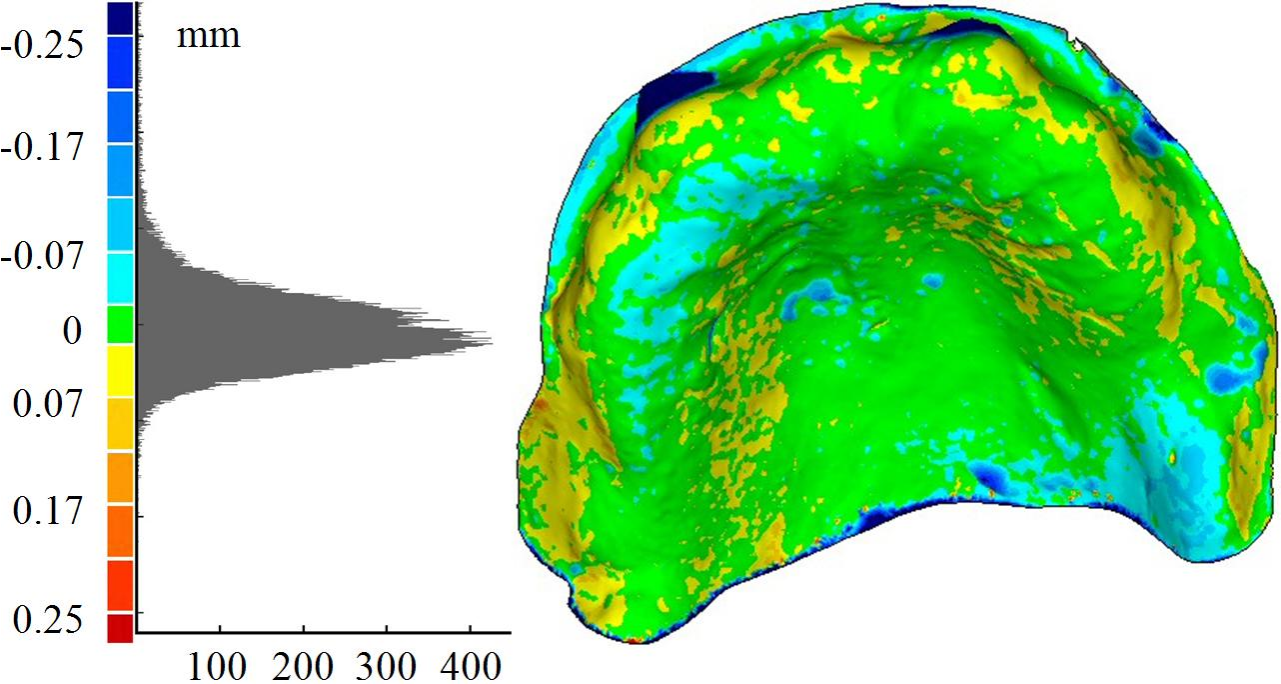
**Full-field 3D comparison between impression and cast**. 3D compare between the impression and the gypsum cast models obtained by optical scanning.

Figure 8 shows the 3D compare between the patient mouth's impression (Figure 7A) and the relative study cast (*mean value *-0.004 mm, *SD *0.067 mm). The manufacturing of the gypsum cast is the first critical step of the whole process that can be verified, since the accuracy in detecting the impression is not measurable. Mismatch between the impression and the gypsum cast may cause improper fitting of the radiographic template, which could result stable on the cast, but floating or not wearable in the patient's mouth.

**Figure 9:**
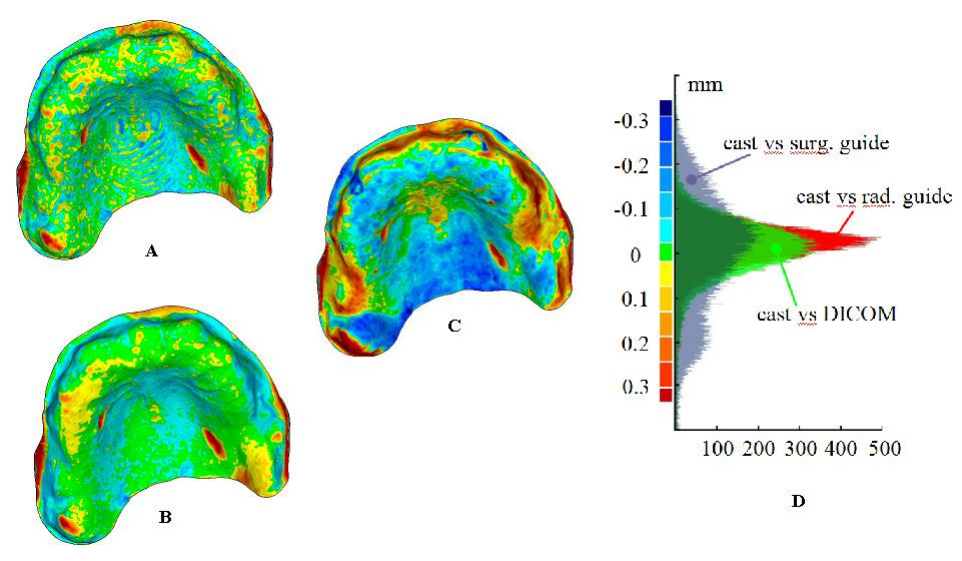
**(A-D) Full-field 3D comparisons between cast and dental models**. Full-field distributions of the measurements discrepancies between gypsum cast model and, respectively, the radiological guide model as obtained by DICOM processing (A), the radiological guide model (B) and the Duplicate radiographic Template "D.R.T." (C) as obtained by the optical scanner. (D) Relative histogram plots.

In Figure 9, the distributions of the optical measurement discrepancies between corresponding points of the gypsum cast and, respectively, the radiological guide (Figure 9B) (*mean value *-0.009 mm, *SD *0.069 mm) and the surgical guide or Duplicate Radiographic Template (Figure 9C) (*mean value *0.013 mm, *SD *0.141 mm) are reported. Moreover, the fitting of the radiological guide model, obtained by processing DICOM images on the gypsum cast has been verified (Figure 9A) (*mean value *-0.004 mm, *SD *0.082 mm). Table 2 summarizes the same results in terms of mean value and standard deviation of the misalignments. Histogram plots relative to these distributions are reported in Figure 9D.

**Table 2:** Statistical data relative to discrepancies between cast and dental models

3D Compare	Impression		Gypsum cast	
	*Mean value* [mm]	*SD* [mm]	*Mean value* [mm]	*SD* [mm]
DICOM	-	-	-0.004	0.082
Gypsum cast	-0.004	0.067	-	-
Radiological template	-	-	-0.009	0.069
Surgical template	-	-	0.013	0.141

Mean and standard deviation of the discrepancies reported in Figure 8 and Figure 9

## Discussion

The analysis of the results allows the detection of possible errors occurred in manufacturing surgical guides. Low discrepancy values between the impression and cast models prove the correctness in the manufacturing process of the gypsum cast. The almost perfect superimposition between the radiological template and the study cast should have been expected since the radiological template is customized by manually fitting it on the cast. The transfer from the radiological to the surgical guides involves two distinct processes: the reconstruction of the radiological guide model by CT scanning and the manufacturing of the surgical guide starting from this digital model. The accuracy of the first step has been verified aligning the model obtained by processing the DICOM images with the gypsum cast. The fine adjustment of the threshold value in the segmentation process, using the model obtained by optical scanning as the anatomical truth, has allowed the minimization of the deviations with respect to the cast. For this reason, the high misalignment errors regarding the surgical template can be attributed to the stereo-lithographic process, which has been used to manufacture the surgical guide. The geometrical differences of the surfaces mating with the gypsum cast, certainly affect the overall accuracy in the implant placement positions. As a further proof, the surgical guide has demonstrated to improperly fit the physical model of the dental gypsum cast. This could lead the surgeon to anchor the template in the wrong way, compromising the desired implant placement.

A thorough study of the effect of these discrepancies on the maximum deviations obtained between the planned positions of the implants and the postoperative result should be done.

## Conclusions

In this paper, a methodology to evaluate the transfer accuracy of CT dental information into periodontal surgical field has been proposed. The procedure is based on the integration of a structured light vision system within the CT scan based preoperative planning process. The use of the optical scanner, having a higher resolution and accuracy than CT scanning, has demonstrated to be a valid support to evaluate the precision of the various physical models adopted and to point out possible error sources. Optical scanning of the radiological guide, mounted on the gypsum cast, could be furthermore helpful for the integration of the prosthetic data within the bone structure. In case of not fully edentulous patients, the acquisition of teeth's shape could be used, in addition to gutta-percha markers, to optimize or verify the positioning of the radiological guide with respect to the maxillofacial structure. Moreover, the accurate digital model of the mouth impression could be the base for the direct design of the radiological guide using CAD/CAM technologies, without passing through manufacturing the gypsum cast, drastically reducing errors and planning time.

## Competing interests

The authors declare that they have no competing interests.

## Authors' contributions

GF, GC, SB, AP, AR and FF participated to the conception and design of the work, to the acquisition of data, wrote the paper, participated in the analysis and interpretation of data and reviewed the manuscript. All the authors read and approved the final manuscript.

## Pre-publication history

The pre-publication history for this paper can be accessed here:

http://www.biomedcentral.com/1471-2342/11/5/prepub

## References

[B1] Vercruyssen M Jacobs R Van Assche N van Steenberghe D The use of CT scan based planning for oral rehabilitation by means of implants and its transfer to the surgical field: a critical review on accuracy J Oral Rehabil 2008 35 454 474 10.1111/j.1365-2842.2007.01816.x 18429973

[B2] Scarfe WC Farman AG Sukovic P Clinical applications of cone-beam computed tomography in dental practice J Can Dent Assoc 2006 72 75 80 16480609

[B3] Tardieu PB Vrielinck L Escolano E Computer-assisted implant placement. A case report: treatment of the mandible Int J Oral Maxillofac Implants 2003 18 599 604 12939016

[B4] Verstreken K Van Cleynenbreugel J Martens K Marchal G van Steenberghe D Suetens P An image-guided planning system for endosseous oral implants IEEE Trans Med Imaging 1998 17 842 852 10.1109/42.736056 9874310

[B5] Eggert DW Lorusso A Fischer RB Estimating 3-D rigid body transformations: a comparison of four major algorithms Mach Vis Appl 1997 9 272 290 10.1007/s001380050048

[B6] Azari A Nikzad S Computer-assisted implantology: historical background and potential outcomes - a review Int J Med Robotics Comput Assist Surg 2008 4 95 104 10.1002/rcs.188 18348182

[B7] Van Assche N van Steenberghe D Guerrero ME Hirsch E Schutyser F Quirynen M Jacobs R Accuracy of implant placement based on pre-surgical planning of three-dimensional cone-beam images: a pilot study J Clin Periodontol 2007 34 816 821 10.1111/j.1600-051X.2007.01110.x 17716317

[B8] Sarment DP Sukovic P Clinthorne N Accuracy of implant placement with a stereolithographic surgical guide Int J Oral Maxillofac Implants 2003 18 571 577 12939011

[B9] Di Giacomo GA Cury PR de Araujo NS Sendyk WR Sendyk CL Clinical application of stereolithographic surgical guides for implant placement: preliminary results J Periodontol 2005 76 503 507 10.1902/jop.2005.76.4.503 15857088

[B10] Al-Harbi SA Sun AY Implant placement accuracy when using stereolithographic template as a surgical guide: preliminary results Implant Dent 2009 18 46 56 10.1097/ID.0b013e31818c6a50 19212237

[B11] Mi W Beirowski B Gillingwater TH Adalbert R Wagner D Grumme D Osaka H Conforti L Arnhold S Addicks K The slow Wallerian degeneration gene, WldS, inhibits axonal spheroid pathology in gracile axonal dystrophy mice Brain 2005 128 405 416 10.1093/brain/awh368 15644421

[B12] Romaniello A Cruccu G Frisardi G Arendt-Nielsen L Svensson P Assessment of nociceptive trigeminal pathways by laser-evoked potentials and laser silent periods in patients with painful temporomandibular disorders Pain 2003 103 31 39 10.1016/S0304-3959(02)00347-0 12749956

[B13] Barone S Paoli A Razionale AV INGEGRAF An Innovative Methodology for the Design of Custom Dental Prostheses by Optical Scanning Proceedings of XXI INGEGRAF: 10-12 June 2009; Lugo 2009 Lugo 264 272

[B14] 3D Slicer (version 3.2) http://www.slicer.org

[B15] Brown AA Scarfe WC Scheetz JP Silveira AM Farman AG Linear accuracy of cone beam CT derived 3D images Angle Orthod 2009 79 150 157 10.2319/122407-599.1 19123719

[B16] Besl PJ McKay ND A Method for Registration of 3D Shapes IEEE Trans Pattern Anal Mach Intell 1992 14 239 256 10.1109/34.121791

